# Changes in Ethylene, ABA and Sugars Regulate Freezing Tolerance under Low-Temperature Waterlogging in *Lolium perenne*

**DOI:** 10.3390/ijms22136700

**Published:** 2021-06-22

**Authors:** Barbara Jurczyk, Ewa Pociecha, Franciszek Janowiak, Michał Dziurka, Izabela Kościk, Marcin Rapacz

**Affiliations:** 1 Department of Plant Breeding, Physiology and Seed Science, University of Agriculture in Kraków, Podłużna 3, 30-239 Kraków, Poland; ewa.pociecha@urk.edu.pl (E.P.); iza.koscik@gmail.com (I.K.); marcin.rapacz@urk.edu.pl (M.R.); 2 The Franciszek Górski Institute of Plant Physiology, Polish Academy of Sciences, Niezapominajek 21, 30-239 Kraków, Poland; f.janowiak@ifr-pan.edu.pl (F.J.); m.dziurka@ifr-pan.edu.pl (M.D.)

**Keywords:** ABA, cold acclimation, ethylene, freezing tolerance, perennial ryegrass, waterlogging

## Abstract

Plant overwintering may be affected in the future by climate change. Low-temperature waterlogging, associated with a predicted increase in rainfall during autumn and winter, can affect freezing tolerance, which is the main component of winter hardiness. The aim of this study was to elucidate the mechanism of change in freezing tolerance caused by low-temperature waterlogging in *Lolium perenne*, a cool-season grass that is well adapted to a cold climate. The work included: (i) a freezing tolerance test (plant regrowth after freezing); (ii) analysis of plant phytohormones production (abscisic acid [ABA] content and ethylene emission); (iii) measurement of leaf water content and stomatal conductance; (iv) carbohydrate analysis; and (v) analysis of *Aco*1, *ABF*2, and *FT*1 transcript accumulation. Freezing tolerance may be improved as a result of cold waterlogging. The mechanism of this change is reliant on multifaceted actions of phytohormones and carbohydrates, whereas ethylene may counteract ABA signaling. The regulation of senescence processes triggered by concerted action of phytohormones and glucose signaling may be an essential component of this mechanism.

## 1. Introduction

Extreme weather events are expected to occur more frequently owing to climate change. Increases in the frequency, intensity, and amount of heavy precipitation events are predicted at high latitudes of the Northern Hemisphere and potentially raise the possibility of flooding [1,2]. The increase in autumn and winter precipitation as well as winter warming events leading to snow melt may change the risk of low-temperature waterlogging. Under such conditions, a plant’s overwintering capability is uncertain [3].

Hypoxic or even anoxic conditions are the primary constraint associated with flooding. The risk of oxygen deficiency in waterlogged soil increases under high temperatures [4]. This phenomenon is associated with the increase in metabolic activity and decrease in water solubility of oxygen. As the oxygen concentration declines slowly in cold water, plants have an extended period in which they can adapt to harsh conditions. Most previous studies of submergence and waterlogging were conducted under relatively high temperatures. However, the physiological, biochemical, and molecular adaptations to waterlogging may be inadequate under different temperatures.

Phytohormones interact and play an important role in hypoxia response and waterlogging tolerance. Ethylene (ET) and auxin crosstalk plays an important role in plants survival under waterlogging [5] by regulating adventitious roots formation. Given physical entrapment and enhanced biosynthesis, the ET content increases in flooded plants, which is an early signal (priming factor) for regulation of the plant’s response to flooding [6]. Ethylene interacts with other phytohormones, including abscisic acid (ABA). Both ET and ABA are responsible for stomatal closure under waterlogging stress [7]. During hypoxia, ET accumulation down-regulates ABA by inhibiting enzymes involved in ABA biosynthesis and activating ABA degradation [8]. This observation may be indicative of an antagonistic relationship between these two phytohormones under flooding.

Abscisic acid may substitute as a low-temperature signal and regulates many of the genes associated with freezing tolerance. The promoter of some cold-regulated genes contains ABA-responsive elements (ABREs) that are induced by ABRE-binding factors (AREB/ABFs) [9]. A member of the ABF family, ABF2 (Abscisic acid responsive elements-binding factor 2), may interact with CBF3 (a C-repeat binding factor, which is a crucial transcription factor involved in cold acclimation) [10] and ABF2 overexpression affects tolerance of multiple stresses [11]. Therefore, cold signal transduction interplays with ABA. However, ET biosynthesis and signaling negatively regulate cold signaling by repressing the expression of cold-inducible CBFs [12]. Cold stress alters ET levels in many plant species, but both increases and reduction in ET levels have been reported [13]. The role of ET in freezing tolerance may be species-dependent. Given its function as a repressor of plant growth and development, ET may be implicated in growth cessation, which is an important aspect of cold acclimation.

Many processes regulated by phytohormones, including those under unfavorable environmental conditions, may also be modulated by sugars, namely glucose and sucrose [14]. Under abiotic stresses, in addition to serving osmoprotective functions, sugars act as signaling molecules engaged in the activation of many stress-inducible genes. Sugars perform a crucial role in crosstalk between different abiotic stress pathways. Some cold-regulated genes are induced by both ABA and sugars [15]. The interaction between sugar signaling and ABA signaling contributes to senescence regulation under abiotic stress [16,17]. Precocious senescence may be initiated as a component of the acclimation response to flooding [18].

Abiotic stresses disturb sugar homeostasis. Possible sugar starvation due to anaerobic respiration under waterlogging may induce storage carbohydrate mobilization to support plant regrowth after freezing. Stress-related phytohormones (ABA, ET) regulate enzymes that participate in fructan biosynthesis (fructosyltranserases; FTs) and catabolism (fructan exohydrolases; FEHs) [19]. However, the interaction mechanisms between different hormones remain largely undetermined. The present study aimed to test the hypothesis that soil waterlogging during cold acclimation can improve freezing tolerance as a result of concerted action of phytohormones (ABA and ET) and changes in carbohydrates. The study was focused on perennial ryegrass (*Lolium perenne*), a cool-season grass that is well adapted to a cold climate. The species is extensively grown as a forage and turf grass, but its winter hardiness is limited when compared to other forage grasses. Here, the mechanism for the change in freezing tolerance in response to cold waterlogging in *L*. *perenne* is explored, which provides insight into this little-known aspect of plant overwintering in a changing climate.

## 2. Results

### 2.1. Freezing Tolerance

**Figure 1:**
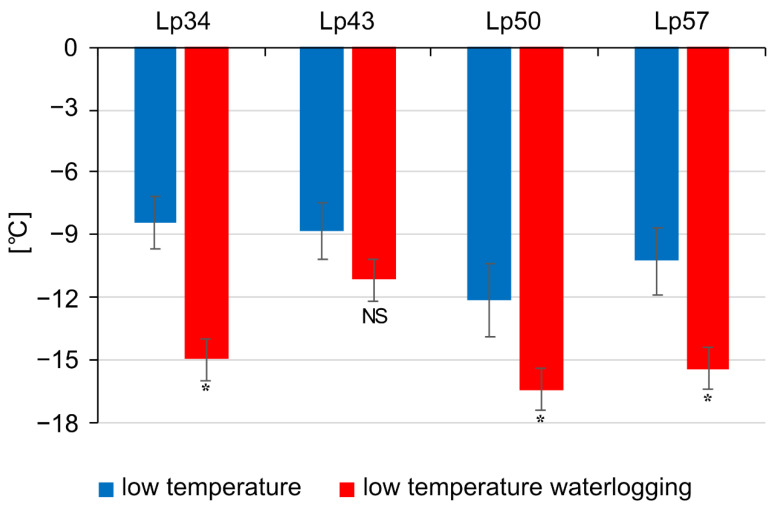
Frost tolerance expressed as the RT_50_ value (the temperature at which a 50% decrease in regrowth is observed) in *Lolium perenne* genotypes cold-acclimated for 21 days. Four genotypes (Lp34, Lp43, Lp50, and Lp57) were tested. The results are means (*n* = 6), error bars indicate the standard error. Asterisks indicate means that differed significantly between waterlogged and control plants (*p* < 0.05).

Estimation of the predicted temperature at which regrowth is reduced by 50% (RT_50_) confirmed that waterlogging during cold acclimation can improve plant freezing tolerance. Three genotypes of *L. perenne* showed an increase in freezing tolerance under waterlogging (Figure 1). Approximately 6.6, 4.3, and 5.2 °C (corresponding to 78%, 36%, and 50%) increases were observed in the genotypes Lp34, Lp50, and Lp57, respectively. No statistically significant change in RT_50_ was observed for Lp43.

### 2.2. Ethylene Emission

**Figure 2:**
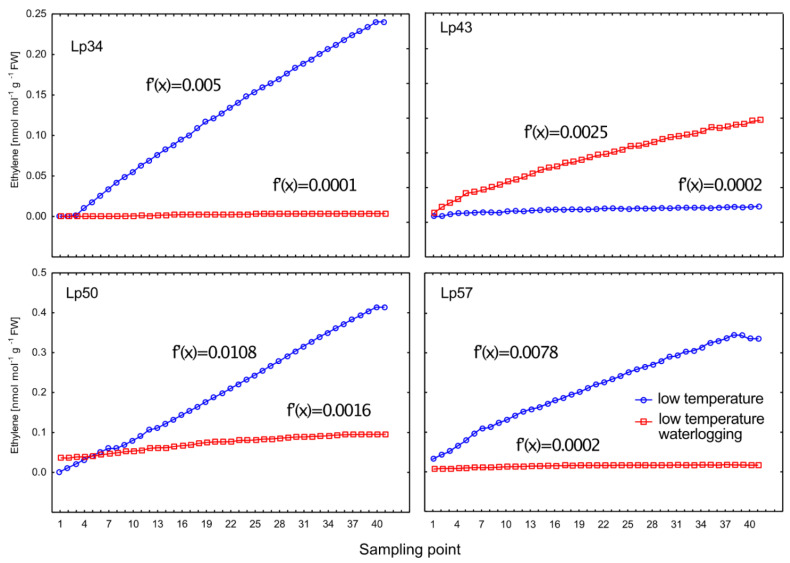
Ethylene accumulation in 2 dm^3^ glass cuvettes after 3 weeks of cold acclimation in waterlogged and control plants of *Lolium perenne*. Measurements were recorded at 1 min 10 s intervals (41 sampling points) and flow rate of 250 cm^3^/min. Four genotypes (Lp34, Lp43, Lp50, and Lp57) were tested. Function derivatives [*f*′(*x*)], describing the ethylene rate of change, are presented.

In genotypes that showed an increase in freezing tolerance under waterlogging (Lp34, Lp50, and Lp57), a lower rate of ET emission was observed in waterlogged plants relative to that of the control (Figure 2). This difference was illustrated by the lower function derivatives for waterlogged plants in relation to that of control plants. After approximately 48 min of measurement of ET accumulation (the duration of the measurement period), the highest ET concentration was observed in the Lp50 genotype control (more than 0.4 nmol/mol/g fresh weight [FW]) and the Lp57 genotype control (more than 0.3 nmol/mol/g FW). During this period, in the Lp34 genotype control, ET emission was slightly less than 0.25 nmol/mol/g FW. In the waterlogged Lp43 genotype, the ET release rate was higher than that of the control (higher function derivatives), with the last measurement exceeding 0.1 nmol/mol/g FW. The Lp43 genotype showed no increase in RT_50_ in response to waterlogging.

### 2.3. Stomatal Conductance

**Figure 3:**
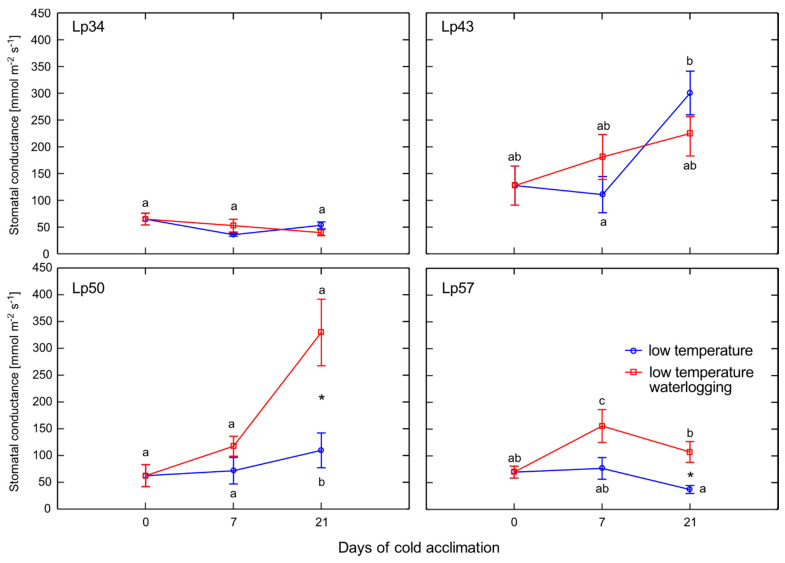
Stomatal conductance of cold-acclimated *Lolium perenne* genotypes. Four genotypes (Lp34, Lp43, Lp50, and Lp57) were tested. The results are means (*n* = 9), error bars indicate the standard error. Asterisks indicate means that differ significantly between waterlogged and control plants according to one-way ANOVA *(p* < 0.05). Different lower-case letters indicate means that are statistically different according to Duncan’s multiple range test (*p* < 0.05).

Stomatal conductance was measured to clarify if changes in ET emission in response to waterlogging may be associated with changes in stomatal conductivity. In particular, we examined whether the observed decreases in ET emission were a result of stomatal closure. No differences in stomatal conductance were observed in waterlogged plants of Lp34 and Lp43 relative to the control (Figure 3). Increases in stomatal conductance were observed in waterlogged plants relative to the control after 21 days of cold acclimation in the Lp50 and Lp57 genotypes. Therefore, the decreased ET release rate in response to waterlogging in Lp34, Lp50, and Lp57 (the genotypes that showed increased freezing tolerance) was not a consequence of stomatal closure. In addition, the increased ET release rate caused by waterlogging in Lp43 was not associated with increased stomatal conductivity.

### 2.4. Water Content

**Figure 4:**
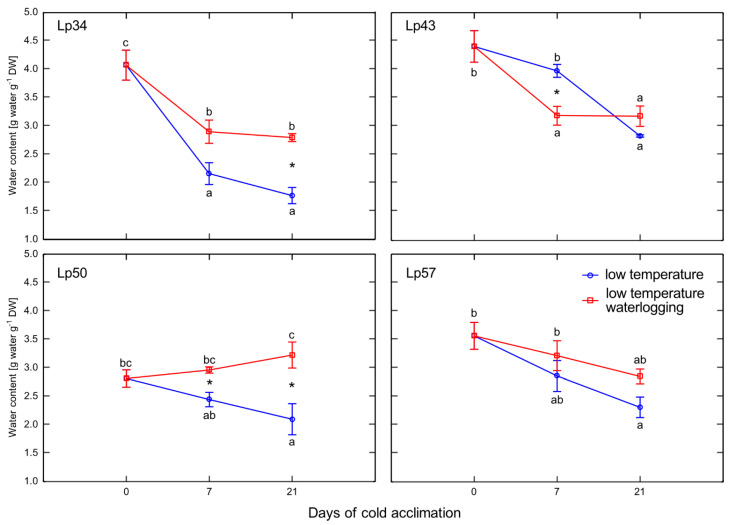
Water content of cold-acclimated *Lolium perenne* genotypes. Four genotypes (Lp34, Lp43, Lp50, and Lp57) were tested. The results are means (*n* = 4), error bars indicate the standard error. Asterisks indicate means that differ significantly between waterlogged and control plants according to one-way ANOVA (*p* < 0.05). Different lower-case letters indicate means that are statistically different according to Duncan’s multiple range test (*p* < 0.05).

Increases in water content were observed in response to the treatment (waterlogging) in the Lp34 genotype after 21 days of cold acclimation and in the Lp50 genotype after 7 and 21 days of cold acclimation (Figure 4). In contrast, for the Lp43 genotype, a decrease in water content was observed after 7 days of cold acclimation in waterlogged plants relative to that of the control. 

### 2.5. ABA Content

**Figure 5:**
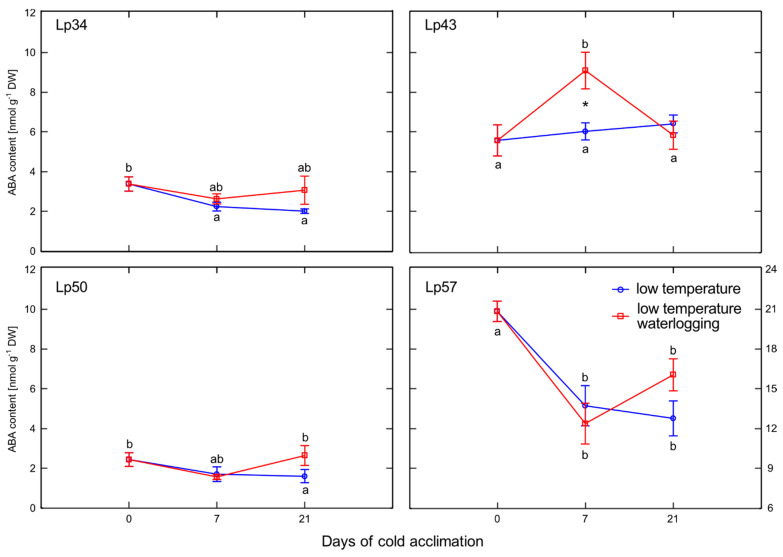
Abscisic acid (ABA) content of cold-acclimated *Lolium perenne* genotypes. Four genotypes (Lp34, Lp43, Lp50, and Lp57) were tested. The results are means (*n =* 4), error bars indicate the standard error. Asterisks indicate means that differ significantly between waterlogged and control plants according to one-way ANOVA (*p* < 0.05). Different lower-case letters indicate means that are statistically different according to Duncan’s multiple range test (*p* < 0.05).

Increase in ABA content was observed in waterlogged Lp43 plants relative to that of the control after 7 days of cold acclimation (Figure 5). This change was a consequence of a transient increase (between 0 and 7 days of the experiment) in ABA content of waterlogged plants. This ABA increase coincided with the decrease in water content detected in waterlogged Lp43 plants. In the other three genotypes, no changes in ABA content were observed in response to waterlogging.

### 2.6. Glucose content

**Figure 6:**
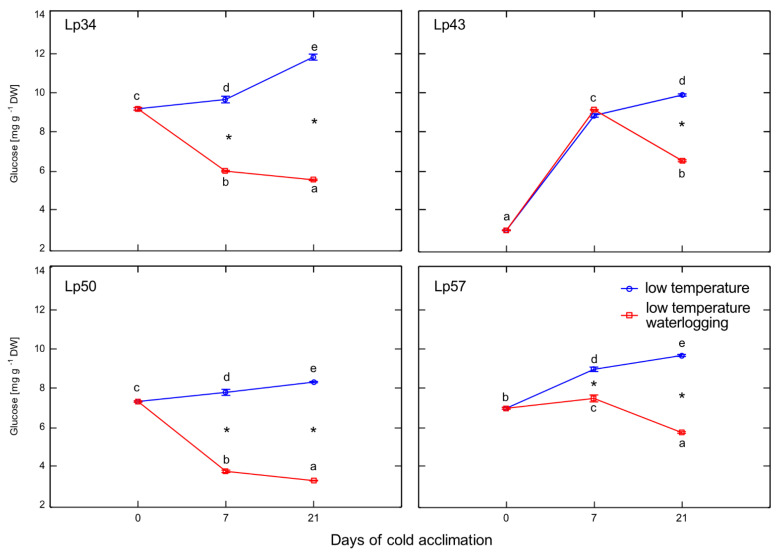
Glucose content of cold-acclimated *Lolium perenne* genotypes. Four genotypes (Lp34, Lp43, Lp50, and Lp57) were tested. The results are means (*n* = 4), error bars indicate the standard error. Asterisks indicate means that differ significantly between waterlogged and control plants according to one-way ANOVA (*p* < 0.05). Different lower-case letters indicate means that are statistically different according to Duncan’s multiple range test (*p* < 0.05).

Decreases in glucose content in response to waterlogging were observed in Lp34, Lp50, and Lp57 plants after 7 and 21 days of cold acclimation, and in Lp43 plants after 21 days of cold acclimation (Figure 6). A relatively high glucose content in Lp43 after 7 days of cold acclimation coincided with an increase in ABA content. Therefore, induction of ABA accumulation might be associated with a relatively high glucose content.

### 2.7. Sucrose Content

**Figure 7:**
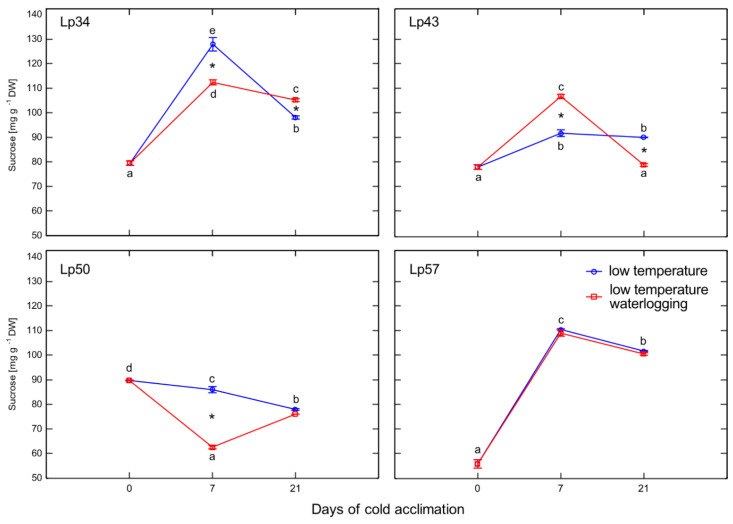
Sucrose content of cold-acclimated *Lolium perenne* genotypes. Four genotypes (Lp34, Lp43, Lp50, and Lp57) were tested. The results are means (*n* = 4), error bars indicate the standard error. Asterisks indicate means that differ significantly between waterlogged and control plants according to one-way ANOVA (*p* < 0.05). Different lower-case letters indicate means that are statistically different according to Duncan’s multiple range test (*p* < 0.05).

Decreases in sucrose content of waterlogged plants relative to that of the control were observed in Lp34 and Lp50 plants after 7 days of cold acclimation, and in Lp43 plants after 21 days of treatment (Figure 7). Increases in sucrose content in response to waterlogging were detected after 21 days of cold acclimation in Lp34 plants and after 7 days of cold acclimation in Lp43 plants. The increase in sucrose content may reflect degradation of fructan polymers into smaller compounds because sucrose is the end-product of this process.

### 2.8. Fructan Content and Polymerization

**Figure 8:**
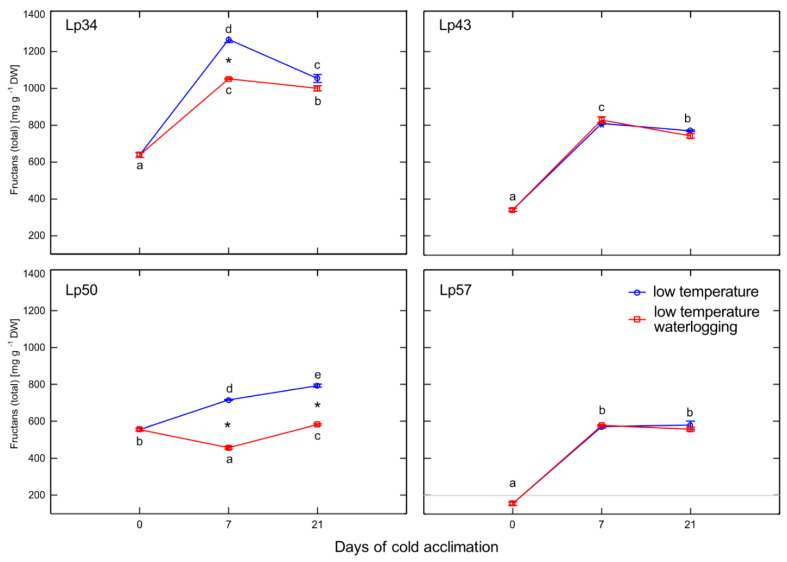
Total fructan content of cold-acclimated *Lolium perenne* genotypes. Four genotypes (Lp34, Lp43, Lp50, and Lp57) were tested. The results are means (*n* = 4), error bars indicate the standard error. Asterisks indicate means that differ significantly between waterlogged and control plants according to one-way ANOVA (*p* < 0.05). Different lower-case letters indicate means that are statistically different according to Duncan’s multiple range test (*p* < 0.05).

In all plants, regardless of the treatment, the total fructan content increased after cold acclimation relative to that of the non-acclimated control. However, the total fructan content was decreased in waterlogged plants relative to that of the control in Lp34 after 7 days of the experiment and in Lp50 after 7 and 21 days of the experiment (Figure 8).

**Figure 9:**
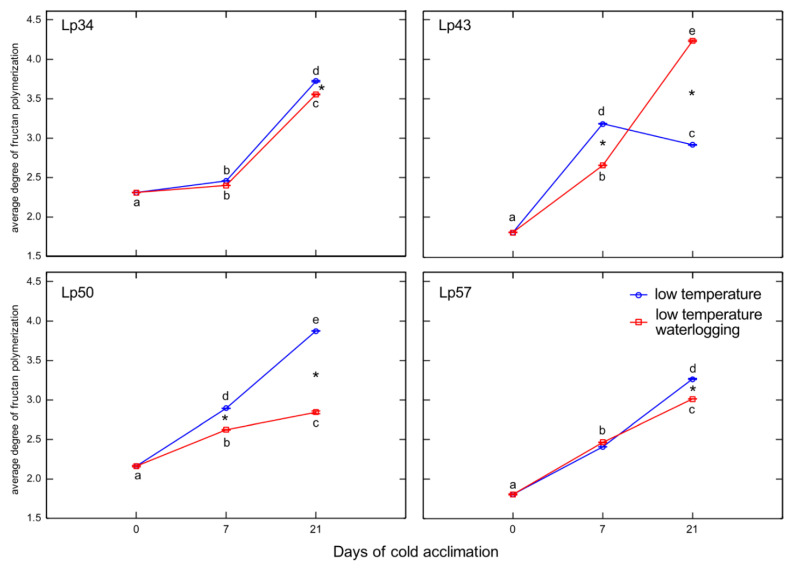
Average degree of polymerization of total fructans of cold-acclimated *Lolium perenne* genotypes. Four genotypes (Lp34, Lp43, Lp50, and Lp57) were tested. The results are means (*n* = 4), error bars indicate the standard error. Asterisks indicate means that differ significantly between waterlogged and control plants according to one-way ANOVA (*p* < 0.05). Different lower-case letters indicate means that are statistically different according to Duncan’s multiple range test (*p* < 0.05).

An increase in fructan polymerization after cold acclimation relative to the non-acclimated control was detected in all plants regardless of the treatment. However, waterlogging may modify the rate of this change. Decreases in the average degree of fructan polymerization in response to waterlogging were observed in Lp50 plants after 7 and 21 days of cold acclimation, in Lp34 and Lp57 after 21 days of cold acclimation, and in Lp43 plants after 7 days of cold acclimation (Figure 9). At the end of the experiment, in Lp43 an increase in fructan polymerization was observed in response to waterlogging.

### 2.9. Aco1 Transcript Level

**Figure 10:**
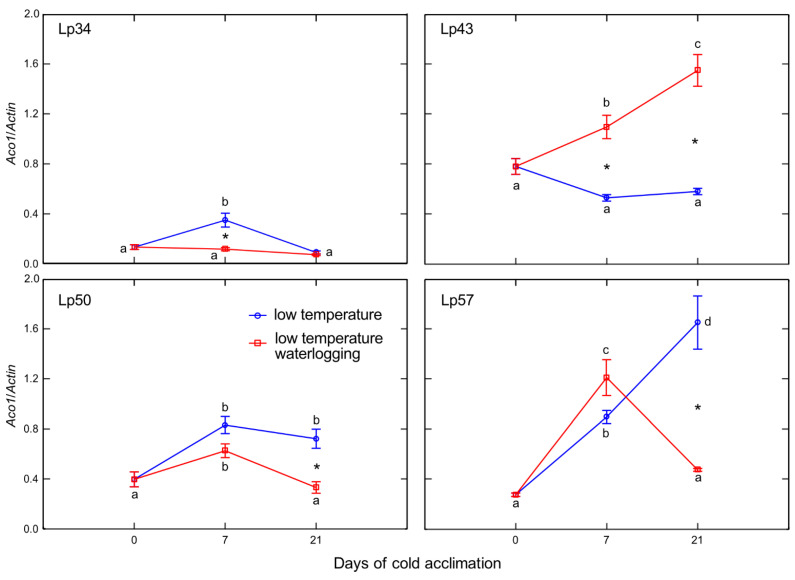
Relative transcript level of *Aco*1 of cold-acclimated *Lolium perenne* genotypes. Four genotypes (Lp34, Lp43, Lp50, and Lp57) were tested. The transcript level was calculated using *Actin* as a reference gene. The results are means (*n* = 4), error bars indicate the standard error. Asterisks indicate means that differ significantly between waterlogged and control plants according to one-way ANOVA (*p* < 0.05). Different lower-case letters indicate means that are statistically different according to Duncan’s multiple range test (*p* < 0.05).

Decreases in the *Aco*1 (*Aminocyclopropane-1-carboxylate oxidase* 1) transcript level in response to waterlogging were detected in Lp34 after 7 days of cold acclimation, and in Lp50 and Lp57 after 21 days of cold acclimation (Figure 10). In Lp43, an increase in *Aco*1 transcript level caused by waterlogging was observed after cold acclimation for 7 and 21 days. The decrease in transcript level of *Aco*1 supports evidence for decreased ET biosynthesis (and subsequently ET emission) under waterlogging at low temperature in genotypes that showed an increase in freezing tolerance. Similarly, the increase in *Aco*1 transcript level under waterlogging in Lp43 (which showed no increase in freezing tolerance) corresponded with the increased rate of ET emission of this genotype.

### 2.10. ABF2 Transcript Level

**Figure 11:**
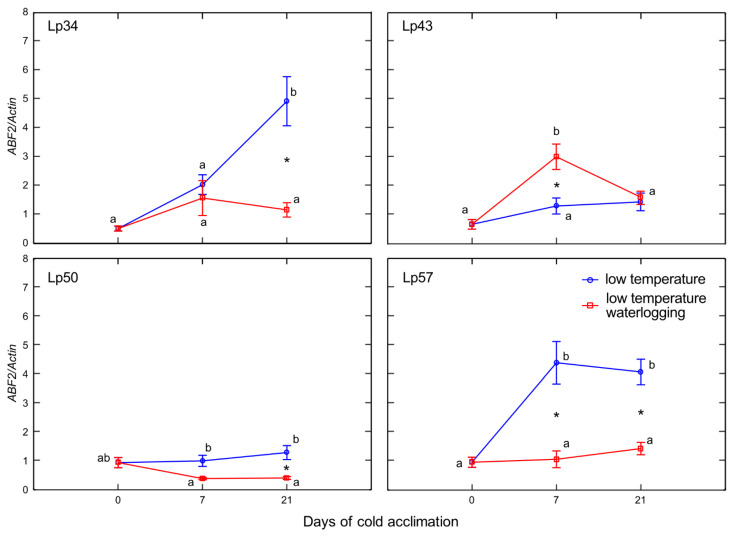
Relative transcript level of *ABF*2 of cold-acclimated *Lolium perenne* genotypes. Four genotypes (Lp34, Lp43, Lp50, and Lp57) were tested. The transcript level was calculated using *Actin* as a reference gene. The results are means (*n* = 4), error bars indicate the standard error. Asterisks indicate means that differ significantly between waterlogged and control plants according to one-way ANOVA (*p* < 0.05). Different lower-case letters indicate means that are statistically different according to Duncan’s multiple range test (*p* < 0.05).

Decreases in *ABF*2 transcript level in response to waterlogging were observed in Lp34 plants after 21 days of cold acclimation, and in Lp50 and Lp57 after 7 and 21 days of cold acclimation (Figure 11). In Lp43, an increase in *ABF*2 transcript level was detected in response to waterlogging after 7 days of cold acclimation. This increase corresponded with the coincident elevation in ABA content observed in Lp43 plants. 

### 2.11. FT1 Transcript Level

**Figure 12:**
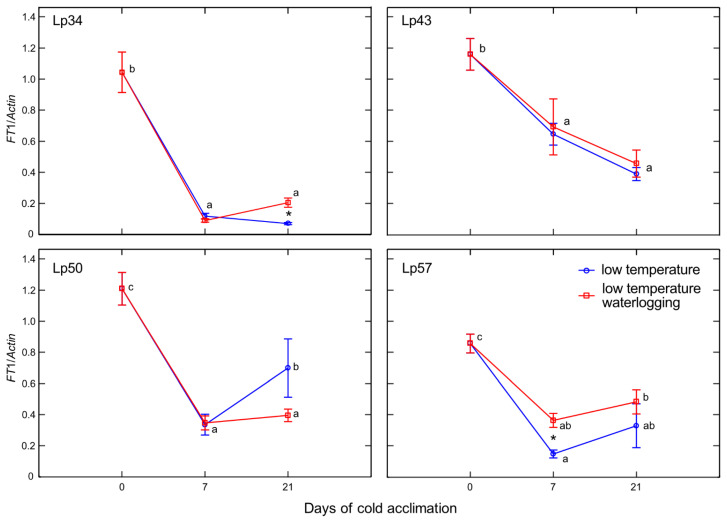
Relative transcript level of *FT1* of cold-acclimated *Lolium perenne* genotypes. Four genotypes (Lp34, Lp43, Lp50, and Lp57) were tested. The transcript level was calculated using *Actin* as a reference gene. The results are means (*n* = 4), error bars indicate the standard error. Asterisks indicate means that differ significantly between waterlogged and control plants according to one-way ANOVA (*p* < 0.05). Different lower-case letters indicate means that are statistically different according to Duncan’s multiple range test (*p* < 0.05).

Increases in the *FT*1 (*Fructosyltransferase* 1) transcript level in response to waterlogging were observed in Lp34 and Lp57 plants after 21 and 7 days of cold acclimation, respectively (Figure 12). In Lp50, a decrease in *FT*1 transcript level was observed in response to waterlogging after 21 days of cold acclimation. This decrease corresponded with a decrease in total fructan content in this genotype. In all genotypes, a decrease in *FT*1 transcript level was detected after cold acclimation.

### 2.12. Principal Component Analysis

**Figure 13:**
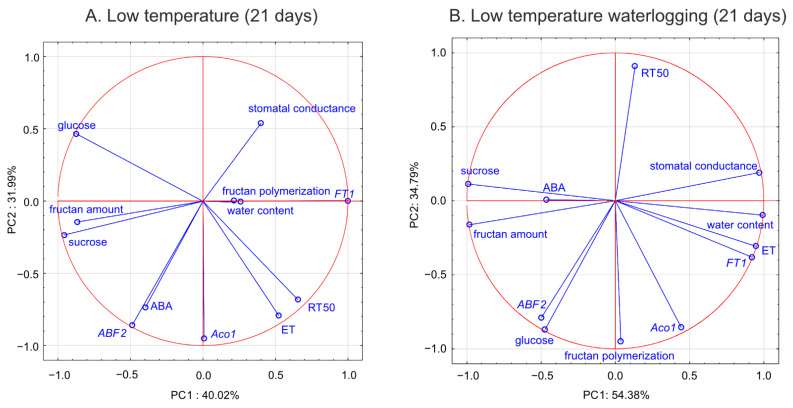
Principal component analysis of analyzed parameters performed in *Lolium perenne* genotypes after 21 days of low temperature (**A**) and low temperature waterlogging (**B**). The first two principal components explained 72.01% and 89.17% of the total variability in the control and waterlogged plants, respectively.

Principal component analysis (PCA) was conducted to explore the relationships between the individual variables and to compare these relationships between treatments. The PCA produced two principal components that explained 72.01% and 89.17% of the total variability in the control and waterlogged plants, respectively (Figure 13). The analysis revealed that the variables showed a distinct response pattern in control versus treated plants. A strong negative relationship between RT_50_ and fructan polymerization was observed in waterlogged plants, and a negative relationship was detected between RT_50_ and *Aco*1 transcript level. In waterlogged plants, a negative relationship was observed between RT_50_ and both *ABF*2 transcript level and glucose content. A negative relationship between ET emission and ABA content was observed in waterlogged plants.

## 3. Discussion

Climatic change will likely affect a plant’s capability for winter survival. However, the impact of climate change on plant overwintering is difficult to predict. Ambiguous results have been reported on the effect of waterlogging on freezing tolerance. These discrepancies originate mainly because of differences in waterlogging pretreatments, in freezing tolerance tests, and, probably most importantly, in species/genotype-dependent tolerance to waterlogging. Thus, neutral, positive, or negative effects of waterlogging on freezing tolerance have been reported [20,21,22,23]. However, the general conclusion can be drawn that waterlogging at higher temperatures is more damaging to plants than waterlogging at lower temperatures [23]. It seems that different waterlogging temperatures may activate different stress-tolerance mechanisms and may lead to entirely different reactions.

In the present study, three of the four genotypes of *L. perenne* showed an increase in freezing tolerance (expressed as plant regrowth after freezing) in response to waterlogging at low temperature. Such observations are in accordance with our previous results [22]. In our previous study, a positive effect of low-temperature waterlogging on plant regrowth after freezing was observed in *L. perenne* and *Festuca pratensis* populations. Freezing tolerance improvement in *F. pratensis* was associated with increased photochemical activity, but the mechanism of freezing tolerance changes in *L. perenne* was not thoroughly explained.

In the current study, ET emission was decreased in genotypes that showed increased freezing tolerance. The increase in ET concentration observed in submerged plants reported to date was mainly associated with physical entrapment rather than increased ET biosynthesis [24]. In the present experiment, plants were waterlogged to approximately 2 cm above the soil surface, therefore, ET was not entrapped in the aboveground plant parts that were not submerged. The intensity of ET emission from plant tissues is a function of both ET biosynthesis and the rate of outward diffusion regulated by stomatal conductance. Thus, in this study, two parameters were examined to explain changes in ET emission: the transcript level of *Aco*1 (which encodes an immediate ET precursor) and stomatal conductance. The potential effect of stomatal closure causing decreased ET emission was excluded. A higher proportion of stomata were opened in two of the three genotypes exhibiting decreased ET emission. However, the decrease in *Aco*1 transcript levels provides evidence for decreased ET biosynthesis under waterlogging at low temperature. The function of ET in cold response is not fully understood, but some evidence suggests that ET signaling plays a negative role in freezing stress tolerance. In Arabidopsis, ET biosynthesis and signaling negatively regulate low-temperature signaling (cold acclimation) through the transcriptional control of cold-inducible CBFs [12]. Here, we suggest that decreased ET biosynthesis under cold waterlogging may contribute to improvement in freezing tolerance, limiting ET’s negative role in cold signaling. This interpretation was supported by PCA because *Aco*1 transcription was negatively associated with RT_50_.

Recent studies suggest that plant response to environmental stresses encompasses not only the activation of defense mechanisms, but also modulation of plant growth and development processes that are obviously regulated by ET. As reported in previous studies, which were mostly performed at relatively high temperatures, flooding causes premature senescence, leaf chlorosis, cessation of growth, and reduced plant yield. In the present study, waterlogged plants showed no visible signs of senescence and were larger than control plants with greater shoot biomass (data not shown). Moreover, decrease in ET emission (Lp34, Lp50, and Lp57) suggests that senescence processes were delayed in waterlogged plants. The mechanism of senescence regulation may involve sugar signaling. It was previously shown that hexoses accumulate in senescing leaves and the glucose supply induces changes in gene expression typical of leaf senescence [25]. Therefore, in the present study, delayed plant senescence may be triggered by decreased glucose content, which was observed in response to waterlogging. Consistently, the *L. perenne* genotype that showed increased ET emission in response to waterlogging (and not enhanced freezing tolerance under waterlogging) showed a relatively high glucose content. Therefore, we suggest that both these triggering factors (ET and glucose) act in concert to regulate plant senescence under cold waterlogging. The two factors may act independently or dependently, as ET may affect glucose signaling [26].

Enhanced senescence during abiotic stresses is an adaptive mechanism that has positive consequences, e.g., regulation of the plant water balance. In the present experiment, no decrease in water content was observed in plants with increased freezing tolerance. Accordingly, no changes in ABA content in response to waterlogging were observed among these genotypes. However, decrease in water content was noted in Lp43 plants in response to waterlogging. Coincident increases in ABA content and *ABF*2 transcript level were observed. The induction of ABA accumulation in waterlogged Lp43 plants may be mediated not only by drought stress, but also by a relatively high glucose content. It was previously shown that glucose controls ABA biosynthesis genes and also ABA signaling genes [26]. This finding is consistent with the present PCA of waterlogged plants, which revealed a close relationship between *ABF*2 transcription and glucose content (a relationship not observed in the control plants).

The protein ABF2 is a crucial regulator of ABA-mediated chlorophyll degradation and leaf senescence via the transcriptional activation of senescence-associated genes [27]. In addition, ABF2 is involved in the metabolic arrest of genes involved in energy metabolism [11]. These findings further suggest that the Lp43 genotype showed enhanced senescence, in contrast to the more freezing-tolerant genotypes that showed delayed senescence in response to waterlogging. Plants with delayed senescence tend to show increased tolerance to abiotic stresses [28]. From this point, we conclude that increased freezing tolerance observed in the present study may be associated with delayed senescence triggered by changes in ET, ABA, and sugars. Consequently, PCA showed that freezing tolerance was negatively associated with glucose content, *ABF*2 transcription, and *Aco*1 transcription.

Abscisic acid is an important signal in the plant response to abiotic stresses [29]. Exogenous ABA application leads to increased plant freezing tolerance [30]. In the current study, the increase in ABA content of waterlogged plants was associated with relatively low freezing tolerance and may suggest that a signal counteracted ABA signaling. ABF2 is an essential component of glucose signaling and its overexpression triggers tolerance of multiple stresses, such as dehydration [11]. The active role of ABF2 is suggested by stomatal closure [11]. In the present experiment, despite an increase in the *ABF*2 transcript level (and increase in ABA content), the stomata were prevented from closing. This phenomenon suggested that a signal modifies ABA signaling and responses. That signal might be ET because, as shown previously in Arabidopsis, under drought stress, ET inhibits ABA-induced stomatal closure [31].

Phytohormones, including ABA, are involved in fructan metabolism [19]. A dual role for ABA was proposed; a low concentration of ABA promotes FTs induction, whereas a high ABA concentration induces FEHs. In the present experiment, after 1 week of cold acclimation of the Lp43 genotype, under a relatively low water content, an increase in ABA content together with a decrease in average degree of fructan polymerization were observed in waterlogged plants relative to those of the control. Such findings suggest that ABA induced FEHs during the response period. ABA can regulate FEHs at the transcriptional level [32]. The decrease in degree of fructan polymerization in the current experiment was confirmed by the increased accumulation of sucrose, the end-product of this process. Such breakdown of larger fructans to smaller and more osmotically active compounds has been observed in wheat plants under drought stress [33]. After 3 weeks of cold acclimation/waterlogging in the same genotype (Lp43), ABA content decreased while the average degree of fructan polymerization increased. As a result, at the end of the experiment (when the freezing tolerance was assessed), Lp43 showed an increased degree of fructan polymerization in waterlogged plants relative to that of control plants. This response seems to be an ET counteraction to ABA function. Ethylene signaling may repress FEH function, thereby reducing glucose and sucrose contents [19]. Sucrose and glucose contents both decreased in Lp43 during the experiment. In the other three genotypes, lower degrees of fructan polymerization were observed in waterlogged plants relative to that of the control when the freezing tolerance was measured. As no increase in fructan content was observed in response to waterlogging, a reduction in high-molecular-weight fructans occurred. Such breakdown of high-molecular-weight fructans to smaller and more osmotically active compounds may prevent dehydration during freezing stress and confer improvement in freezing tolerance. This seems to be a common strategy in *L. perenne* genotypes, because all waterlogged plants showed a decrease in degree of fructan polymerization, but this reaction was transient in the genotype with relatively low freezing tolerance.

Temperate grasses show higher fructan content when grown at low temperature (5–10 °C) compared with that under warm temperatures (15–25 °C) [34]. This trend is in accordance with the present results showing an increase in total fructan content after cold acclimation relative to that of the non-acclimated control. Waterlogging during cold acclimation may impair or modify the response shown by the Lp34 and Lp50 genotypes. Such conditions are obviously more energy consuming. In response to waterlogging, the total fructan content was decreased relative to the control in Lp34 (which showed a transient decrease) and in Lp50 (for both cold acclimation terms). It seems that fructan biosynthesis supported the fructan content (an energy reserve) in waterlogged Lp34 and Lp57 plants because *FT*1 expression was increased in these genotypes. The decrease in fructan content in Lp50 was partly due to decreased biosynthesis, because *FT*1 expression decreased in response to waterlogging. However, the amount of fructan reserves under waterlogging was not critical for the plant’s freezing tolerance because the genotype that showed a decrease in fructan content relative to the non-waterlogged control exhibited enhanced freezing tolerance. The role of fructans in cold acclimation and in plant tolerance to low temperature remains unclear. Thorsteinsson et al. [35] concluded that the function of fructan biosynthesis during a low-temperature period is to provide a carbohydrate sink to maintain a high photosynthetic rate. Here, the sink activity was increased in response to waterlogging as illustrated by the decrease in glucose content. This finding is in accordance with Valluru and Van Ende [36], who concluded that the optimal mixture of high and low degree of fructans polymerized with hexoses and sucrose, not the fructan content, provides superior protection under freezing stress. This conclusion was confirmed by the present PCA, which showed that not the fructan content, but the degree of fructan polymerization, was associated with RT_50_.

**Figure 14:**
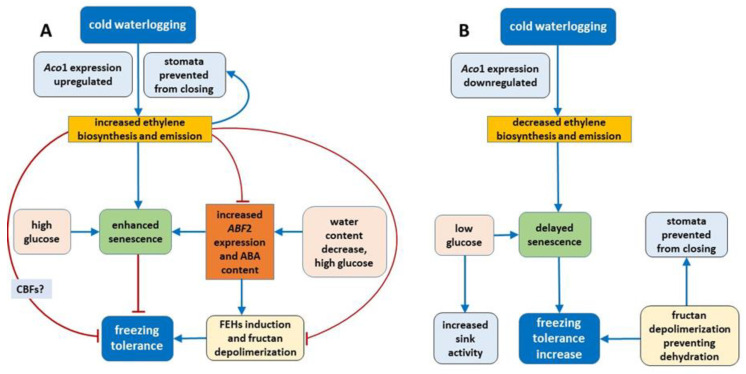
Proposed mechanism of change in freezing tolerance under cold waterlogging in *Lolium perenne* (**A**) genotypes that showed no freezing tolerance increase; (**B**) genotypes that increased freezing tolerance. Blue lines represent positive regulation. Red lines indicate negative regulation.

On the basis of the present results, we can conclude that the mechanism of change in freezing tolerance changes under waterlogging at low temperature relies on the concerted action of phytohormones (ET and ABA) and carbohydrates (Figure 14).

(1) Regulation of some parameters related to senescence (ET, ABA, ABF2 and glucose) plays a role in freezing tolerance under cold waterlogging.
(2) Under cold waterlogging, a high ET concentration counteracts ABA signaling to determine plant freezing tolerance.
(3) Fructan depolymerization (triggered by ABA induced FEHs or other mechanism) provides the optimal fructan mixture, which may be an additional factor that regulates freezing tolerance.
(4) ET may also control cold-inducible CBFs, which is another possible mechanism of freezing tolerance regulation, but this suggestion needs further verification.

## 4. Materials and Methods

### 4.1. Plant Materials and Stress Treatment

**Figure 15:**
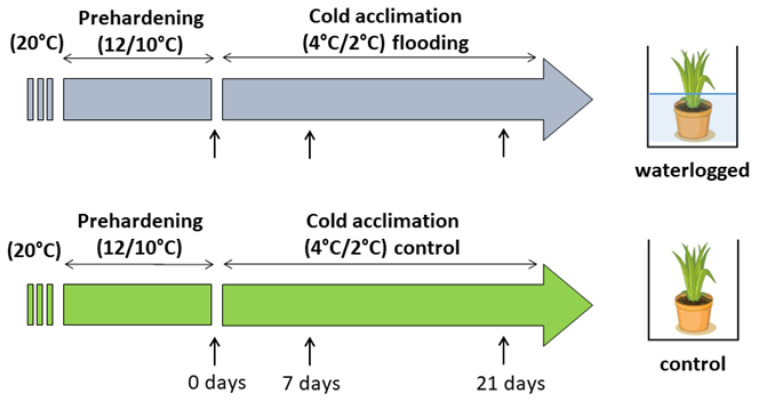
Schematic illustration of the experimental design and treatments. Sampling time points are indicated by arrows.

The experiment was conducted on clones of four *L*. *perenne* ‘Arka’ genotypes (Lp34, Lp43, Lp50, and Lp57). Clones for each genotype were raised from a single seed and were selected from a larger population of *L*. *perenne* genotypes (Lp50 and Lp57 were more freezing tolerant, and Lp34 and Lp43 were less freezing tolerant). Seed sowing, germination, and plant growth before treatment were performed under controlled conditions in a growth chamber (20 °C, 10 h/14 h [light/dark] photoperiod), with light provided by high-pressure sodium (HPS) lamps (Philips) of 300 µmol m^−2^ s^−1^ photosynthetic photon flux density (PPFD), as described by Jurczyk et al. [37]. Seedlings were cultivated in 20-cm-diameter pots containing a mixture of soil, sand, and peat in equal volumes and were irrigated optimally. The clones were divided into smaller portions when they comprised more than approximately 100 tillers. When the plants were 24 months old, each genotype was represented by 10–12 clones and during that time all plants were subjected to prehardening (14 days, 12/10 °C, 10 h/14 h photoperiod, and 300 µmol m^−2^ s^−1^ PPFD provided by the HPS lamps). After the prehardening stage, plants were subjected to cold acclimation (21 days at 4/2 °C, 10 h/14 h photoperiod, and 300 µmol m^−2^ s^−1^ PPFD provided by the HPS lamps). During the first day of cold acclimation (in the early morning), half of the clones (five to six clones) were waterlogged using tap water in plastic boxes to maintain the water level at 2–3 cm above the soil level. The water level was monitored during the experiment and water was refilled when necessary. Control plants were put in similar plastic boxes but irrigated optimally. The measurements were recorded after prehardening (before waterlogging, 0 days) and after 7 and 21 days of cold acclimation from waterlogged and control plants (Figure 15). The replicates were taken from four to six clones of each genotype per treatment.

### 4.2. Freezing Tolerance

Freezing tolerance tests for each genotype were conducted in a freezing chamber. The temperatures for the tests were selected based on previous results and the experience of the authors. Cold-acclimated plants of each genotype were tested at four temperatures: −6, −8, −10, or −12 °C. The plants were placed in a freezing chamber initially at a temperature of 2 °C in the dark [38]. The temperature was lowered by 3 °C/h until the required frost level was attained, and then the plants were kept at this temperature for 6 h. The temperature was raised subsequently to 2 °C at the rate of 3 °C/h. Then the plants were decapitated 4 cm above the soil surface and transferred to a growth chamber to observe regrowth at 12 °C under an 8 h/16 h (light/dark) photoperiod and 150 μmol m^−2^ s^−1^ PPFD. 

After 21 days, plant regrowth was estimated visually using a 0–9 point scale: 0—a completely dead plant with no signs of leaf growth; 1—plants with leaf elongation of about 0.5 cm before dying; 2—dying plants with a leaf elongation of about 1–2 cm; 3—dying plants with leaf elongation of more than 2 cm; 4—plants that may die or may grow but the inner youngest leaves are brown; 5—plant that may survive but damage is visible and the regrowing leaves are discolored and curled; 6—the plant survived but damage is visible on about 50% of the leaves; 7—the plant survived, but symptoms of freezing injury are visible on some of the leaves, which are discolored or deformed; 8—only the tips of some of the inner youngest leaves are discolored or deformed; and 9—no symptoms of injury.

Based on the frost injuries observed in the temperature range from −6 to −12 °C, the predicted temperature at which regrowth is reduced by 50% (RT_50_) was determined for each genotype. The value of 4.5—half of the maximum value 9—was assumed for 50% of the regrowth reduction. The RT_50_ value was estimated, on the basis of six independent replicates, from a linear regression fitted to the sigmoid relationship between the freezing temperatures and the regrowth score using the four tested temperatures.

### 4.3. Ethylene measurement

Ethylene emission was measured in real-time with an electrocatalytic ethylene sensor (EASI-1, Absoger, France). Whole plants without any wounding ET as a result of cutting were individually transferred into closed glass cuvettes (2 dm^3^). The root system with the soil block was tightly closed in a plastic bag so as not to measure ethylene of soil origin produced by micro-organisms. Measurements were recorded in a ventilated room at ambient temperature in the continuous analysis mode at a time interval of 1 min 10 s and the flow rate of 250 cm^3^/min. After each measurement, the cuvettes were ventilated. The ethylene sensor was checked before each use, and calibration with standard gas was performed if necessary. After the completion of measurements, the linear regression line was calculated and the derivative function [*f*′(*x*)] was calculated to estimate the rate of change of ET emission in both waterlogged and control plants.

### 4.4. Leaf Stomatal Conductance

Perennial ryegrass, as a member of the *Poaceae*, bears amphistomatous leaves with stomata on both the upper and lower leaf surfaces [39,40]. However, our preliminary measurements showed that, under the growth conditions used in the experiments, a much higher number of stomata were observed on the upper leaf surface. Thus, the stomatal aperture of leaf surfaces was assessed based on the measurements on the adaxial surface of leaves using a diffusion porometer (AP4, Delta-T Devices, Cambridge, UK) and expressed as stomatal conductance (mmol m^−2^ s^−1^). For each treatment, nine measurements were performed on nine leaves of five to six clones. 

### 4.5. ABA Content

Plant samples were freeze-dried and ground with a ball mill (MM400, Retsch, Haan, Germany) in Eppendorf vials, to which 1.5 mL cold distilled water was then added. The vials were heated for 3 min in a thermoblock set to 90 °C and then shaken overnight at 4 °C to extract ABA [41]. The next day, the aqueous extracts were centrifuged for 20 min at 18,000 × *g* in a refrigerated centrifuge (MPW-350R, Warsaw, Poland). The ABA content was measured in the supernatant using an indirect enzyme-linked immunosorbent assay (ELISA) in accordance with the procedure of Walker-Simmons & Abrams [42]. The antibody used was MAC 252 (Babraham Technix, Cambridge, UK). Absorbance was measured at 405 nm with a microplate reader (Model 680, Bio-Rad Laboratories, Hercules, CA, USA). Four independent pool samples, each consisting of leaves from five to six clones, were collected and after FW determination were immediately frozen. At least two ELISA measurements were performed for each sample, yielding a total of six ABA measurements for each treatment.

### 4.6. Leaf Water Content

Leaf water content (LWC) was estimated based on the leaf fresh weight (FW) and leaf dry weight (DW) after freeze-drying the samples collected for ABA measurement and expressed as g water per g DW.

### 4.7. Carbohydrate Analysis

Sugars were analyzed in accordance with a modified method reported by Hura et al. (2016). Measurements were performed on four independent pool samples, each consisting of leaves from five to six clones. Harvested plant material was preserved in liquid nitrogen, then lyophilized in a freeze-dryer (LGA05, MLW, Leipzig, Germany, upgraded by JWE, Warsaw, Poland), and homogenized in a mixer mill (MM 400, Retsch, Haan, Germany). Exactly weighted samples (about 15 mg) were added to 1 mL ultra-pure water, heated for 5 min at 80 °C, and extracted for 15 min with shaking at 30 Hz (MM 400, Retsch) at ambient temperature. Samples were centrifuged (15 min at 21,000 × *g*), the supernatant was collected, and extraction was repeated. The combined supernatants were aliquoted into two portions; one was diluted with acetonitrile (1:1, *v*/*v*) and analyzed by high-performance liquid chromatography (HPLC) for soluble sugar content, and the second was used for estimation of the fructan pool.

#### 4.7.1. Fructan Pool and Average Degree of Fructan Polymerization

The fructan pool was estimated after enzymatic hydrolysis of the sugar extract. A mixture of exo-inulase and endo-inulinase supplied by Megazyme (Bray, Ireland) was used. A sample (200 µL) of the carbohydrate extract was diluted with 200 mM sodium acetate buffer (1:1, *v*/*v*, pH 4.5), mixed with 100 µL enzymatic preparation, and diluted with 100 mM sodium acetate buffer (1:5, *v*/*v*, pH 4.5). Samples were hydrolyzed overnight at 40 °C and then the suspension was diluted with acetonitrile (1:1, *v*/*v*), centrifuged, and analyzed by HPLC. The average degree of polymerization (DP_av_) of the fructans was calculated in accordance with the formula of Verspreet et al. [43]:

DP_av_ = 1 + (*F*_f_/*G*_f_),

where *F*_f_ and *G*_f_ are molar concentrations of fructose and glucose released after enzymatic cleavage of fructans. The interference of free glucose, fructose, sucrose, raffinose, and kestoses was determined by subtracting their glucose and fructose equivalents from the total glucose and fructose pool measured after enzyme hydrolysis.

#### 4.7.2. HPLC Analysis of Sugars

Free sugars and fructo-oligosaccharides hydrolyzate were analyzed by HPLC using an Agilent 1200 binary system (Agilent, Wolbrum, Germany) coupled to an electrochemical detector (Coulochem II, ESA, Chelmsford, MA, USA). Soluble sugars (glucose, fructose, sucrose, and other fructans) were separated on a RCX-10 column (7 µm, 250 mm × 4.1 mm; Hamilton, Reno, NV, USA) in gradient mode of 75 mM NaOH solution, and 500 mM sodium acetate in 75 mM NaOH solution, at 1.5 mL/min. Pulsed amperometric detection was employed (analytical potential 200 mV, oxidizing potential 700 mV, and reducing potential −900 mV with reference to a palladium electrode) on a gold electrode. Further technical details are given by Hura et al. [44].

#### 4.7.3. *Aco*1, ABF2, and FT1 Transcripts Levels

Quantitative PCR analysis using QuantStudio 3 (Thermo Fisher Scientific, Waltham, MA, USA) was performed to calculate changes in the relative transcript level of *Aco*1, *ABF*2, and *FT*1. The *Actin* gene was used as an endogenous control gene [45]. Samples were collected after prehardening and after 7 and 21 days of cold acclimation from waterlogged and control plants and were frozen in liquid nitrogen. Approximately 0.04 g of the central portion of mature leaves were used for RNA isolation with RNeasy Plant Mini Kit (Qiagen, Hilden, Germany) in accordance with the manufacturer’s protocol. Complementary cDNA synthesis together with genomic DNA elimination was performed using the QuantiTect Reverse Transcription Kit (Qiagen) in accordance with the manufacturer’s protocol. Approximately 500 ng RNA template was used for each reverse-transcription reaction. The RNA concentration and quality were determined using an UV-Vis Spectrophotometer Q5000 (Quawell, San Jose, CA, USA). The PCR reactions were run in quadruplicate in 96-well plates in a reaction volume of 25 µL, containing 15 µL Power SYBR Green PCR Master Mix, 900 nM each primer, and 25 ng template cDNA. The PCR primers were designed using Primer Express v. 3.0.1 software (Applied Biosystems by Life Technologies, Foster City, CA, USA). The primer sequences and the origin of sequences are presented in Table 1. The PCR amplification used the following protocol: 10 min at 95 °C, 40 cycles of 15 s at 95 °C, and 1 min at 60 °C. After amplification, a dissociation step was added to verify the specificity of the reactions (15 s at 95 °C, 1 h at 60 °C, and 15 s at 95 °C). Data analysis employed QuantStudio Design and Analysis v.1.5.0 software dedicated to the QuantStudio 3 system. To determine the relative transcript levels, the relative standard curve method (Applied Biosystems) was used with *Actin* as an endogenous reference gene. The results presented are the means of four biological replicates taken from four independent plants, each in four PCR technical replicates.

### 4.8. Statistical Analysis

The effect of waterlogging treatment during both cold acclimation terms (7 and 21 days) for a particular genotype was tested using one-way ANOVA (with waterlogging as a factor, *p* = 0.05). The general effects of the treatment were tested using two-way ANOVA (with genotype and waterlogging as factors, *p* = 0.05). Duncan’s multiple range test was employed to determine time-course changes in a particular genotype in waterlogged and control plants separately. Principal component analysis was conducted separately for control and waterlogged plants to visualize relationships between measured variables and the impact of each variable on differentiation of the genotypes. The PCA was conducted by eigenvalue decomposition of a data correlation matrix. The analysis was performed after 21 days of the experiment. All statistical analyses were performed using Statistica v. 13.1 (StatSoft, Tulsa, OK, USA).

## Figures and Tables

**Table 1:** Genes, sequence origins, and designed primers and probes used in the study.

Gene Name	GenBank ID	Forward Primer	Reverse Primer
*ABF*2	MN207248.1	AACATGGACGAGCTGCTCC	GAACTCCTCCAGCGTCATGG
*Aco*1	MN244172.1	TCTTGTACCGGCCGTTGGT	GCCACTCCATCGTCGTCAA
*FT*1	AF481763.1	CACCGCGCCACACAACT	TAGCCAACGTCAGCCTCATTG
*Actin*	AY014279.1	CAAATCATGTTCGAGACCTTCAAT	CACTGGCATAGAGGGAAAGCA

## Abbreviations

ABA	Abscisic acid
ABF2	Abscisic acid responsive elements-binding factor 2
ABRE	ABA-responsive element
*Aco*1	*Aminocyclopropane-1-carboxylate oxidase* 1
AREB	ABRE-binding protein
CBF3	C-repeat binding factor 3
ET	Ethylene
FEHs	Fructan exohydrolases
FTs	Fructosyltransferases
PCA	Principal component analysis

## References

[1] Vautard R., Gobiet A., Sobolowski S., Kjellstrom E., Stegehuis A., Watkiss P., Mendlik T., Landgren O., Nikulin G., Teichmann C. (2014). The European climate under a 2 °C global warming. Environ. Res. Lett..

[2] IPCC (2018). Global Warming of 1.5 °C.

[3] Rapacz M., Ergon A., Hoglind M., Jorgensen M., Jurczyk B., Ostrem L., Rognli O.A., Tronsmo A.M. (2014). Overwintering of herbaceous plants in a changing climate. Still more questions than answers. Plant Sci..

[4] Drew M.C. (1992). Soil aeration and plant root metabolism. Soil Sci..

[5] Qi X., Li Q., Ma X., Qian C., Wang H., Ren N., Shen C., Huang S., Xu X., Xu Q. (2019). Waterlogging-induced adventitious root formation in cucumber is regulated by ethylene and auxin through reactive oxygen species signalling. Plant Cell Environ..

[6] Voesenek L.A.C.J., Sasidharan R. (2013). Ethylene- and oxygen signalling—Drive plant survival during flooding. Plant Biol..

[7] Else M.A., Janowiak F., Atkinson C.J., Jackson M.B. (2009). Root signals and stomatal closure in relation to photosynthesis, chlorophyll *A* fluorescence and adventitious rooting of flooded tomato plants. Ann. Bot..

[8] Schachtman D.P., Goodger J.Q.D. (2008). Chemical root to shoot signaling under drought. Trends Plant Sci..

[9] Uno Y., Furihata T., Abe H., Yoshida R., Shinozaki K., Yamaguchi-Shinozaki K. (2000). *Arabidopsis* basic leucine zipper transcription factors involved in an abscisic acid-dependent signal transduction pathway under drought and high-salinity conditions. Proc. Natl. Acad. Sci. USA.

[10] Lee S.-J., Kang J.-Y., Park H.-J., Kim M.D., Bae M.S., Choi H.-I., Kim S.Y. (2010). DREB2C interacts with ABF2, a bZIP protein regulating abscisic acid-responsive gene expression, and its iverexpression affects abscisic acid sensitivity. Plant Physiol..

[11] Kim S., Kang J.Y., Cho D.I., Park J.H., Kim S.Y. (2004). ABF2, an ABRE-binding bZIP factor, is an essential component of glucose signaling and its overexpression affects multiple stress tolerance. Plant J..

[12] Shi Y., Tian S., Hou L., Huang X., Zhang X., Guo H., Yang S. (2012). Ethylene signaling negatively regulates freezing tolerance by repressing expression of *CBF* and type-A *ARR* genes in *Arabidopsis*. Plant Cell.

[13] Eremina M., Rozhon W., Poppenberger B. (2016). Hormonal control of cold stress responses in plants. Cell. Mol. Life Sci..

[14] Sami F., Yusuf M., Faizan M., Faraz A., Hayat S. (2016). Role of sugars under abiotic stress. Plant Physiol. Biochem..

[15] Masclaux-Daubresse C., Purdy S., Lemaitre T., Pourtau N., Taconnat L., Renou J.-P., Wingler A. (2007). Genetic variation suggests interaction between cold acclimation and metabolic regulation of leaf senescence. Plant Physiol..

[16] Yang J.C., Zhang J.H., Wang Z.Q., Zhu Q.S., Liu L.J. (2003). Involvement of abscisic acid and cytokinins in the senescence and remobilization of carbon reserves in wheat subjected to water stress during grain filling. Plant Cell Environ..

[17] Munne-Bosch S., Lalueza P. (2007). Age-related changes in oxidative stress markers and abscisic acid levels in a drought-tolerant shrub, *Cistus clusii* grown under Mediterranean field conditions. Planta.

[18] Hurng W.P., Lur H.S., Liao C.K., Kao C.H. (1994). Role of abscisic acid, ethylene and polyamines in flooding-promoted senescence of tobacco leaves. J. Plant Physiol..

[19] Valluru R. (2015). Fructan and hormone connections. Front. Plant Sci..

[20] Andrews C.J., Pomeroy M.K. (1981). The effect of flooding pretreatment on cold hardiness and survival of winter cereals in ice encasement. Can. J. Plant Sci..

[21] Dalmannsdottir S., Ostrem L., Larsen A. (2012). Effect of water saturation of soil on winter survival of red clover (*Trifolium pratense*) and timothy (*Phleum pratense*) in Norway. Grassl. A Eur. Resour..

[22] Jurczyk B., Pociecha E., Janowiak F., Kabala D., Rapacz M. (2016). Variation in waterlogging-triggered stomatal behavior contributes to changes in the cold acclimation process in prehardened *Lolium perenne* and *Festuca pratensis*. Plant Physiol. Biochem..

[23] Jorgensen M., Torp T., Molmann J.A.B. (2020). Impact of waterlogging and temperature on autumn growth, hardening and freezing tolerance of timothy (*Phleum pratense*). J. Agron. Crop Sci..

[24] Banga M., Slaa E.J., Blom C., Voesenek L. (1996). Ethylene biosynthesis and accumulation under drained and submerged conditions. A comparative study of two *Rumex* species. Plant Physiol..

[25] Pourtau N., Jennings R., Pelzer E., Pallas J., Wingler A. (2006). Effect of sugar-induced senescence on gene expression and implications for the regulation of senescence in *Arabidopsis*. Planta.

[26] Leon P., Sheen J. (2003). Sugar and hormone connections. Trends Plant Sci..

[27] Gao S., Gao J., Zhu X., Song Y., Li Z., Ren G., Zhou X., Kuai B. (2016). ABF2, ABF3, and ABF4 promote ABA-mediated chlorophyll degradation and leaf senescence by transcriptional activation of chlorophyll catabolic genes and senescence-associated genes in *Arabidopsis*. Mol. Plant.

[28] Rivero R.M., Kojima M., Gepstein A., Sakakibara H., Mittler R., Gepstein S., Blumwald E. (2007). Delayed leaf senescence induces extreme drought tolerance in a flowering plant. Proc. Natl. Acad. Sci. USA.

[29] Kim S.Y. (2007). Recent advances in ABA signaling. J. Plant Biol..

[30] Gilmour S.J., Thomashow M.F. (1991). Cold acclimation and cold-regulated gene-expression in ABA mutants of *Arabidopsis thaliana*. Plant Mol. Biol..

[31] Tanaka Y., Sano T., Tamaoki M., Nakajima N., Kondo N., Hasezawa S. (2005). Ethylene inhibits abscisic acid-induced stomatal closure in *Arabidopsis*. Plant Physiol..

[32] Ruuska S.A., Lewis D.C., Kennedy G., Furbank R.T., Jenkins C.L.D., Tabe L.M. (2008). Large scale transcriptome analysis of the effects of nitrogen nutrition on accumulation of stem carbohydrate reserves in reproductive stage wheat. Plant Mol. Biol..

[33] Virgona J.M., Barlow E.W.R. (1991). Drought stress induces changes in the nonstructural carbohydrate-composition of wheat stems. Aust. J. Plant Physiol..

[34] Chatterton N.J., Harrison P.A., Bennett J.H., Asay K.H. (1989). Carbohydrate partitioning in 185 accessions of Gramineae grown under warm and cool temperatures. J. Plant Physiol..

[35] Thorsteinsson B., Harrison P.A., Chatterton N.J. (2002). Fructan and total carbohydrate accumulation in leaves of two cultivars of timothy (*Phleum pratense* Vega and Climax) as affected by temperature. J. Plant Physiol..

[36] Valluru R., Van den Ende W. (2008). Plant fructans in stress environments. Emerging concepts and future prospects. J. Exp. Bot..

[37] Jurczyk B., Rapacz M., Krepski T. (2015). Photosynthetic Apparatus Responses to Short-term low-temperature Flooding May Contribute to Freezing Tolerance Changes in Forage Grasses. J. Agron. Crop Sci..

[38] Janeczko A., Dziurka M., Pociecha E. (2018). Increased leaf tocopherol and β-carotene content is associated with the tolerance of winter wheat cultivars to frost. J. Agron. Crop Sci..

[39] Willmer C., Fricker M. (1996). Stomata.

[40] Peterson K.M., Rychel A.L., Torii K.U. (2010). Out of the Mouths of Plants: The Molecular Basis of the Evolution and Diversity of Stomatal Development. Plant Cell.

[41] Quarrie S.A., Whitford P.N., Appleford N.E.J., Wang T.L., Cook S.K., Henson I.E., Loveys B.R. (1988). A monoclonal antibody to (S) abscisic acid: Its characterisation and use in a radioimmunoassay for measuring abscisic acid in crude extracts of cereal and lupin leaves. Planta.

[42] Walker-Simmons M.K., Abrams S.R., Davies W.J., Jones H.G. (1991). Use of ABA immunoassays. Abscisic Acid, Physiology and Biochemistry.

[43] Verspreet J., Pollet A., Cuyvers S., Vergauwen R., Van den Ende W., Delcour J.A., Courtin C.M. (2012). A simple and accurate method for determining wheat grain fructan content and average degree of polymerization. J. Agric. Food Chem..

[44] Hura T., Dziurka M., Hura K., Ostrowska A., Dziurka K. (2016). Different allocation of carbohydrates and phenolics in dehydrated leaves of triticale. J. Plant Physiol..

[45] An Y.Q., McDowell J.M., Huang S.R., McKinney E.C., Chambliss S., Meagher R.B. (1996). Strong, constitutive expression of the *Arabidopsis ACT2/ACT8* actin subclass in vegetative tissues. Plant J..

